# Supplementary material for: Principled neuromorphic reservoir computing
Source: Nat Commun. 2025 Jan 14;16:640. doi: 10.1038/s41467-025-55832-y (PMC11733134; doi:10.1038/s41467-025-55832-y)
Supplement: Supplementary file 1 — Supplementary Information [file 41467_2025_55832_MOESM1_ESM.pdf]

Supplementary Material
Principled Neuromorphic Reservoir Computing

This supplementary material first provides the results for the forth (Section S-I) and fifth tasks (Sections S-II), Methods 4.6, following by additional experimental results that are relevant for the distributed representation scheme, Sections S-III-S-VII, as well as, finally, extends the side-by-side evaluation of the product representation and distributed representation schemes by performing the experiments resembling those in the supplementary material in [1], Sections S-VIII-S-X.

S-I Predicting Kuramoto–Sivashinsky system

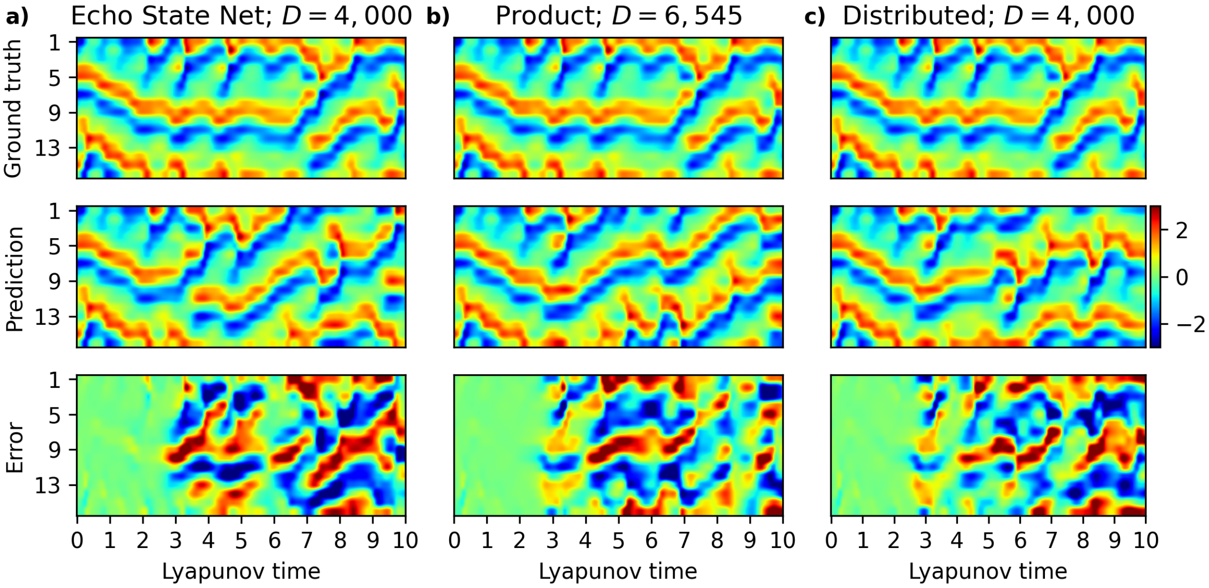

Figure S.1: **Predicting Kuramoto–Sivashinsky system, using a): echo state network, b): product representation and c): distributed representation.** The upper row shows the ground truth for the first 10 Lyapunov times. The central row depicts the corresponding predictions while the errors follow in the lower row. The hyperparameters of the echo state network: $D = 4,000$, $\beta = 0.1$, $\gamma = 0.1$, $\alpha = 1 \times 10^{-8}$. For the product and distributed representations: $k = 2$, $\mathcal{M} = (i, i-1)$, and $\mathcal{T} = (0, 1, 2)$. The regularization parameter for the product representation is set to $\alpha = 1 \times 10^{-4}$, $D = 6,545$. For the distributed representation $\alpha = 1 \times 10^{-6}$ and $D = 4,000$. The corresponding NRMSEs of the depicted single runs are: 6.58×10^{-2}, 7.22×10^{-2}, and 8.91×10^{-2}.

This section presents the results for the fourth task, Methods 4.6. The goal is to evolve the Kuramoto–Sivashinsky system in the autoregressive mode. The results for the echo state network, the product representation, and the distributed representation are reported in Figures S.1 and S.2. Figure S.1 visualizes single runs of the considered schemes. Qualitatively, with sufficient dimensions, they all perform equally well, but note that the echo state network and the distributed representation achieve this performance with smaller reservoirs, $D = 4,000$, while the product representation based on the features up to third-order requires $D = 6,545$. Quantitatively, the median normalized root-mean-square error (NRMSE) across 100 simulations over three Lyapunov times during the prediction phase for the echo state network is 7.76×10^{-1}, for the product representation it is 7.15×10^{-1}, and for the distributed representation it is 8.12×10^{-1}.

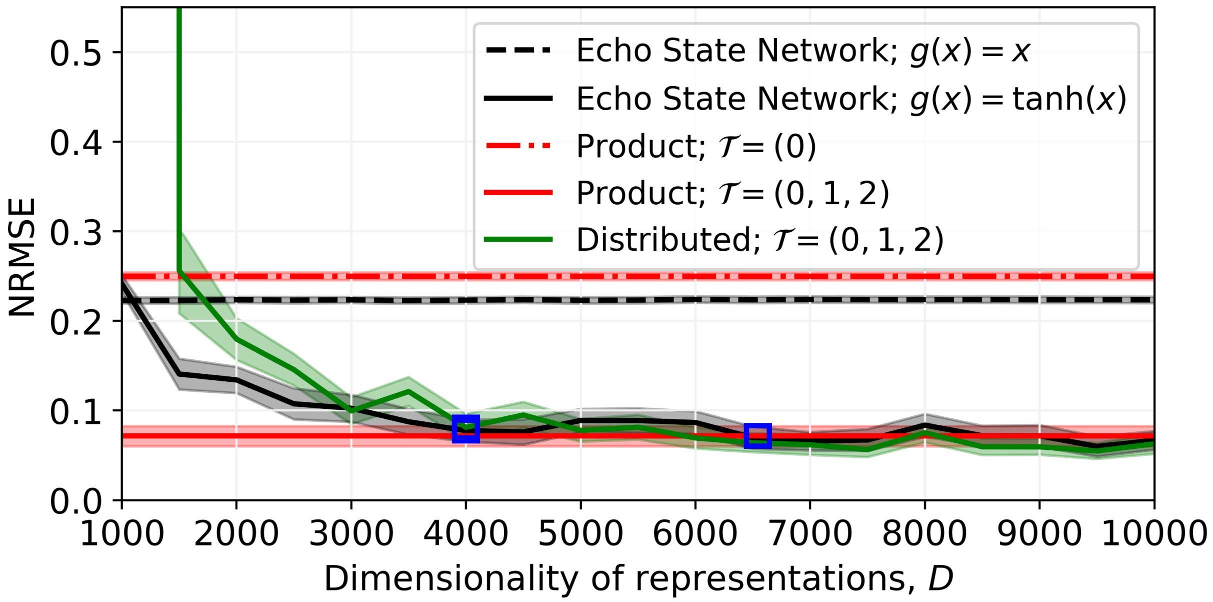

Figure S.2: **Median predictive performance of the echo state network and the distributed representation against D for the Kuramoto–Sivashinsky system.** The the considered models are denoted in the legend. Solid lines correspond to the configurations from Figure S.1. For all configurations of product and distributed representations $k = 2$, $\mathcal{M} = (i, i-1)$. The boxes additionally emphasize the exact location of the configurations reported in Figure S.1 with respect to their reservoirs' sizes. For each configuration, NRMSE is computed over three Lyapunov times where the reported values are obtained from 100 randomly initialized simulations. Shaded areas show the median standard error.

Figure S.2 aims at assessing the effect of the dimensionality of randomized representations on the performance of the echo state network and the distributed representation, as well as investigating the role on nonlinear features on the quality of predictions. In order to assess the importance of nonlinear features, we first trained the models that only consider time-delayed states. For the echo state network, we created the linear reservoir network ($\beta = 0.4$, $\gamma = 0.55$, $\alpha = 1 \times 10^{-7}$) by replacing $\tanh(\cdot)$ activation function in Eq. (22) with the identity function (black dashed line). For the product representation, only the first-order features were considered ($\mathcal{T} = (0)$, red dash-dotted line). The predictive performance of both linear models is equally poor. This part of the experiments highlights that it is essential to consider nonlinear features that go beyond the time-delayed states.

The product and distributed representation schemes provide such nonlinear features as products between the time-delayed states. To achieve good performance with these schemes we had to form the feature space that includes first-, second-, and third- order features, $\mathcal{T} = (0, 1, 2)$, including only first-, and second- order features is not sufficient (not shown). The considered configuration amounts to $D = 6,545$ features for the product representation (red solid line) and provides much better predictive performance than the linear models. When the same feature space is instantiated with the distributed representation (green solid line), approximately equal performance is achieved already at $D = 4,000$ (i.e., about 40% fewer dimensions). For the echo state network, the nonlinear features are formed inside the reservoir through applying $\tanh(\cdot)$ to the random projections of the observable states. Similar to the product representation, using the echo state network with nonlinear features (black solid line) significantly improves upon its linear counterpart. While it performed slightly better than other schemes for the smallest dimensions tested,[1] both the distributed representation and the echo state network achieve the predictive performance of the product representation at about $D = 4,000$ without significant improvements for larger values of D. Thus, for this task both implicit nonlinear features obtained via $\tanh(\cdot)$ activation and explicit polynomial features are equally amenable for obtaining strong predictive performance.

[1]Note that this is consistent with other results, e.g., in Figure S.4 below, which show that higher-order features perform worse than lower-order ones if the dimension is too low.

S-II Missing state prediction

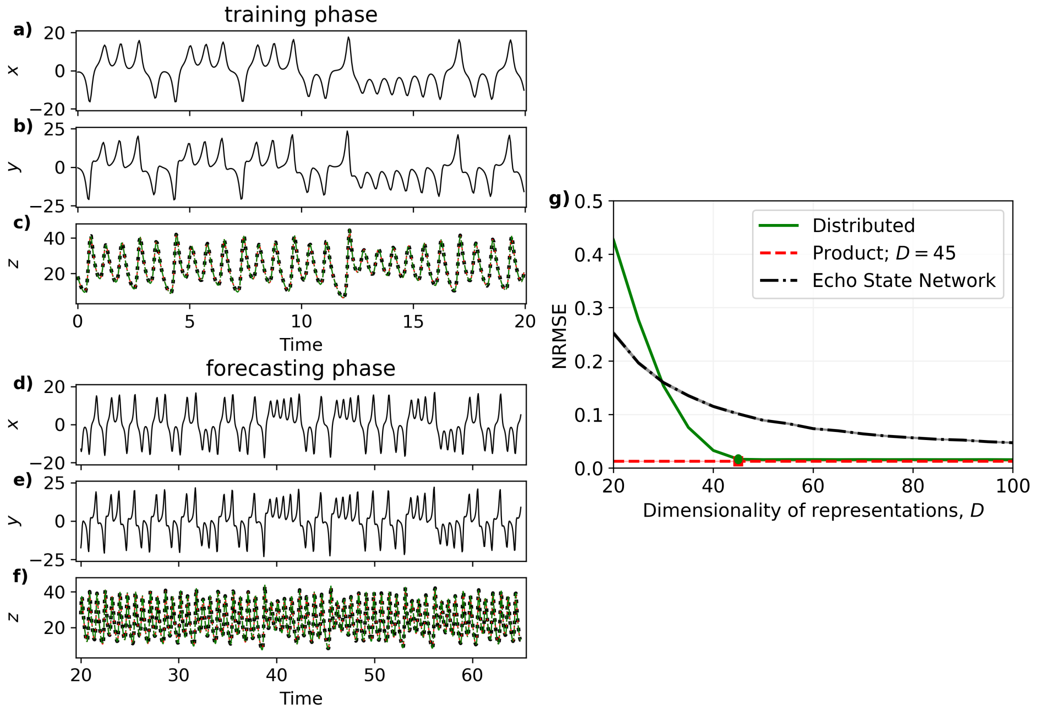

Figure S.3: **Missing state prediction using the product representation, distributed representation, and echo state network schemes.** **a**)–**c**) States of the Lorenz63 system during the training phase (black); the corresponding predictions by product (red dashed line) and distributed (green dash-dotted line) representations are overlayed in **c**). For both schemes the regularization parameter is set to $\alpha = 1 \times 10^{-7}$. **d**)–**f**) States of the Lorenz63 system during the prediction phase. Predictions from both schemes overlay the ground truth in **f**). **g**): Median predictive performance of the distributed representation and the echo state network against the dimensionality of randomized representations D; the distributed representation configuration depicted in panels [**c**) & **f**)] is marked by the green circle. The hyperparameters of the echo state networks: $\beta = 0.4$, $\gamma = 0.85$, and $\alpha = 1 \times 10^{-4}$. The median performance of the corresponding product representation is shown as thick red dashed line where the red square additionally emphasizes the exact location of the configuration with respect to model's size that is driven by features used to form the feature space. For each configuration, the reported NRMSE values are obtained from 1,000 randomly initialized simulations.

This section presents the results for the fifth task, Methods 4.6. As introduced in the task description, the goal is to make one-step-ahead prediction of the dynamics of a state in the Lorenz63 system ($dt = 0.05$) that is non-observable during the prediction phase. The following parameters are used for the product and distributed representations: $d = 2$, $k = 4$, $\mathcal{M} = (i, i-5, i-10, i-15)$ so the total number of features in $\mathbf{G}$ is $1 + 8 + 44 = 45$; for the distributed representation D is set to match this number (but see Figure S.3**g** for the results with various D). As in the first two tasks, the number of training time points is set to $r = 400$, Figure S.3**a-c**, which is sufficient for both schemes to obtain the readout matrix $\mathbf{W}_{\texttt{out}}$ closely following the dynamics of state z during the training phase (overlayed dashed and dash-dotted lines in Figure S.3**c**). During the prediction phase, both the product representation and distributed representation schemes produce high-quality predictions of state z (cf. Figure S.3**f**) from states x and y (Figures S.3**d-e**) resulting in almost the same NRMSE. Thus, this experiment confirms the observation made for the first experiment (cf. Figure 4 in the main text) that, in terms of model size, the distributed representation scheme performs, at least, as well as the product representation one. The additional results reported for the echo state network with the hyperparameters optimized for $D = 45$, Figure S.3**g**, confirm that it can also solve this task but requires a larger reservoir dimension to start producing predictions of quality similar to that of the product or distributed representations.

S-III Predicting the Mackey-Glass system with a network of randomly connected Sigma-Pi neurons

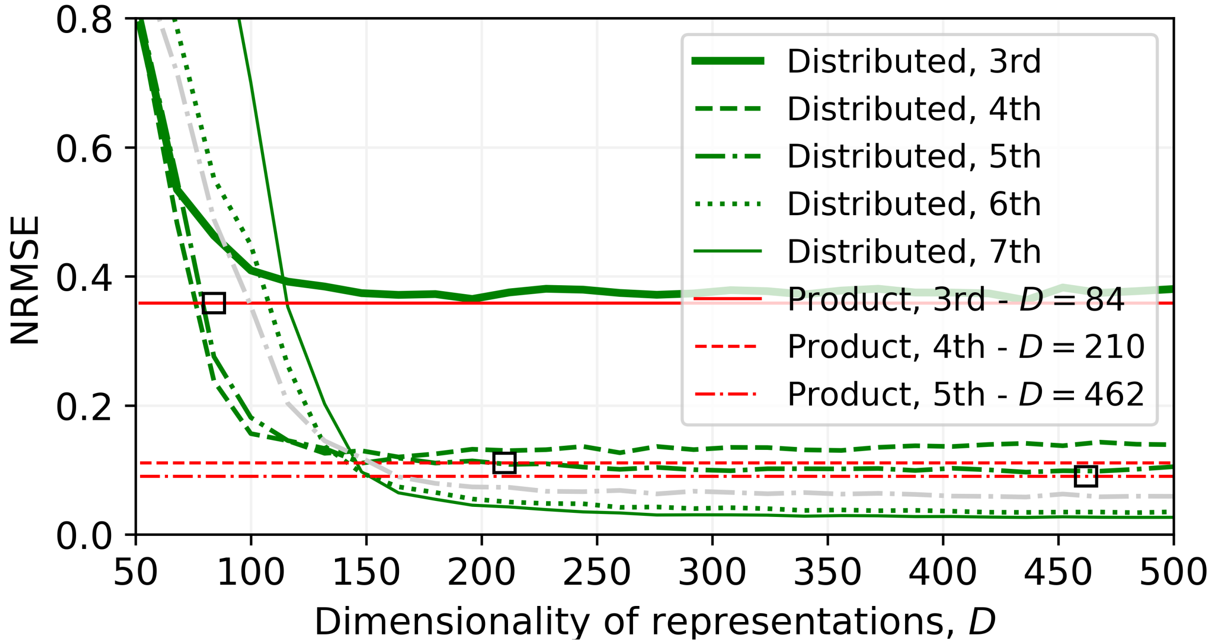

Figure S.4: **Median predictive performance on the Mackey-Glass system using the distributed representation implemented in a network of randomly connected Sigma-Pi neurons against the dimensionality of randomized representations D for models including different largest order of features (see legend).** To provide the baselines, median performance of the product representations using third-, forth-, and fifth-order features are shown by thin solid red, dashed red, and dash-dotted red lines, respectively. Another baseline is provided by the grey dash-dotted line that depicts the results from Figure 4**c** for the structured network with fifth-order features. For each configuration, NRMSE is computed over three Lyapunov times where the reported values are obtained from $1,000$ randomly initialized simulations.

In Section 2.4, we have presented several designs based on a network of Sigma-Pi neurons (cf. Figure 2) that allow efficiently computing distributed representations of higher-order features. Since all of the designs are inspired by particular VSA models with predefined realizations of the binding operation, it is not surprising that each network's connectivity is very structured. How important is the particular structure of the network for obtaining useful representations of higher-order features? Inspired by the findings in [2] where it has been shown that associative memories can be formed using randomly connected Sigma-Pi neurons, we performed the experiments with the setup from Section 2.5 where the design for holographic reduced representations is modified such that instead of the structured connections between Pi and Sigma neurons random connections are used. The sparsity of connections is preserved – each Sigma neuron is receiving inputs from about D Pi neurons that are chosen randomly from the whole population of D^2 Pi neurons. Figure S.4 reports the results. Importantly, the results are closely resembling the results depicted in Figure 4**c** (cf. gray dash-dotted line for the structured network with fifth-order features), hence, the same observations apply, which, in turn, gives an important insight that meaningful computations can be performed even by randomly connected networks of Sigma-Pi neurons.

S-IV Kernel machine implicitly realizes the product representation

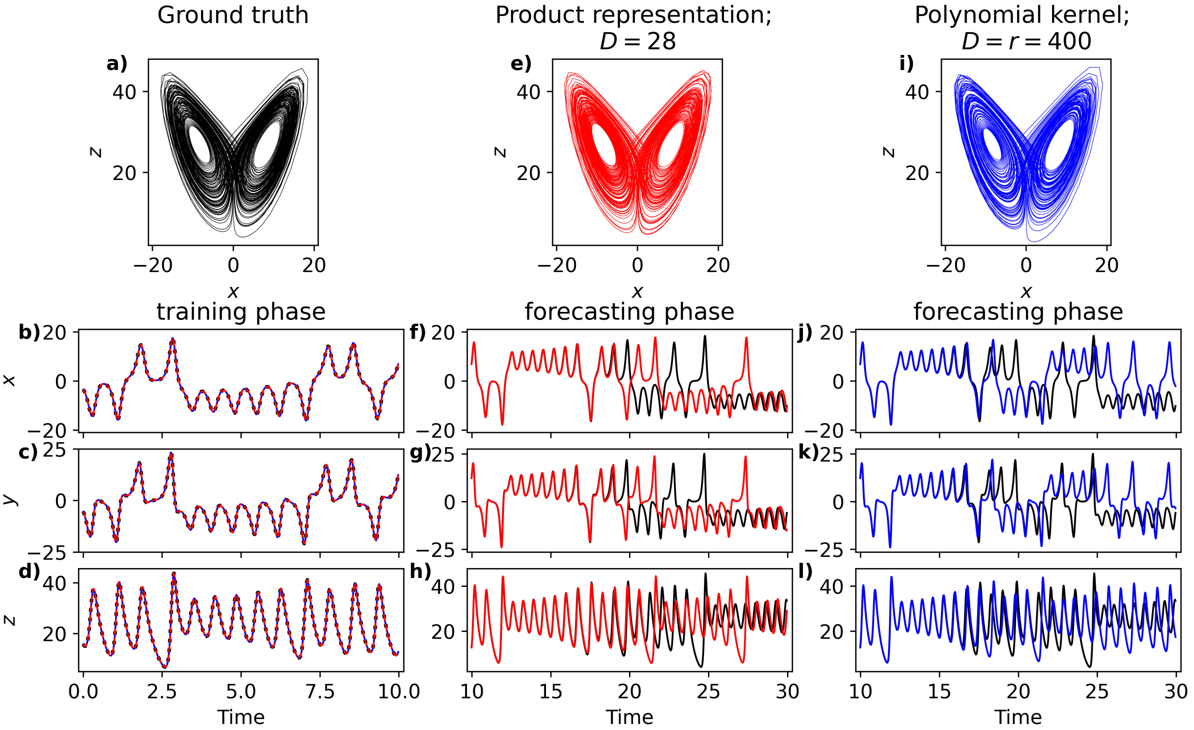

Figure S.5: **Predicting the Lorenz63 dynamical system (left column) using the product representation (central column) or the polynomial kernel machine (right column).** True [**a**)] and predicted [**e**) & **i**)] strange attractors. **b**)–**d**) Training time points (dotted lines) overlayed with the corresponding predictions for the product representation (dashed lines) and the kernel machine (dash-dotted lines) models. For the product representation, regularization parameter is set to $\alpha = 2.5\times10^{-6}$ while for the kernel machine – $\alpha = 1\times10^{-7}$. **f**)–**l**) comparison of the ground truth behavior (black) to the dynamics predicted by either the product representation [**f**)-**h**)] or the kernel machine [**j**)-**l**)]. For the depicted realization of the system, NRMSE over three Lyapunov times during the prediction phase is 4.66×10^{-2} and 3.86×10^{-2}, respectively.

As described in Methods 4.2, the functionality of the product representation can be reinterpreted through the lens of kernel methods. In particular, the representations in $\mathbf{G}$ are nearly identical to the feature map corresponding to the polynomial kernels of various degrees [3] where the kernel functions can be evaluated by simply exponentiating the inner product of the first-order features: $\langle \mathbf{G}^{(1)}(i), \mathbf{G}^{(1)}(j)\rangle^t$. For example, the kernel function $\kappa(\mathbf{G}^{(1)}(i), \mathbf{G}^{(1)}(j))$ corresponding to the feature space used for the Lorenz63 system in Eq. (27) is:

$$\kappa(\mathbf{G}^{(1)}(i), \mathbf{G}^{(1)}(j)) = \langle \mathbf{G}^{(1)}(i), \mathbf{G}^{(1)}(j)\rangle^0 + \langle \mathbf{G}^{(1)}(i), \mathbf{G}^{(1)}(j)\rangle^1 + \langle \mathbf{G}^{(1)}(i), \mathbf{G}^{(1)}(j)\rangle^2, \tag{S.1}$$

while that for the Mackey-Glass system in Eq. (31):

$$\kappa(\mathbf{G}^{(1)}(i), \mathbf{G}^{(1)}(j)) = \sum_{t=0}^{3} \langle \mathbf{G}^{(1)}(i), \mathbf{G}^{(1)}(j)\rangle^t. \tag{S.2}$$

In general, the entries of the kernel matrix $\mathbf{K}$ are computed following Eq. (13). The kernel matrix is then used for obtaining the weights of the training time points in the kernel regression machine, Eq. (14) that, in turn, allows computing the predictions, Eq. (15).

Given an appropriate value of the regularization hyperparameter α, the polynomial kernel machine provides performance equivalent to that of the product representation. To illustrate this point, Figure S.5 presents the results for predicting the Lorenz63 dynamical system, Methods 4.6. Note that panels **a**) & **e**)-**h**) in Figure S.5 are identical to those in Figure 3**a-b** in Section 2.5 of the main text while panels **b**)-**d**) are almost equivalent depicting predictions for the polynomial kernel machine instead of the distributed

representation. Panels **i**)-**l**) present the results obtained with the polynomial kernel machine where the kernel function is evaluated according to Eq. (S.1) and $\alpha = 1 \times 10^{-7}$.

An immediate argument not in favor of the kernel machine interpretation of the product representation is that there are no obvious computational benefits from such an interpretation. For instance for the task of predicting the Lorenz63 system in Figure S.5, the readout matrix in the product representation consists of 84 parameters ($\mathbf{W}_{\mathtt{out}} \in [3 \times 28]$) while the equivalent polynomial kernel machine requires 1200 parameters ($\boldsymbol{\alpha}^{(m)} \in [400 \times 1]$, $m = 3$). However, the kernel machine interpretation provides an important conceptual advantage as it allows thinking about any kind of kernel functions, their feature maps, and approximations of those maps.

S-V Performance for the Lorenz63 system with features of different order

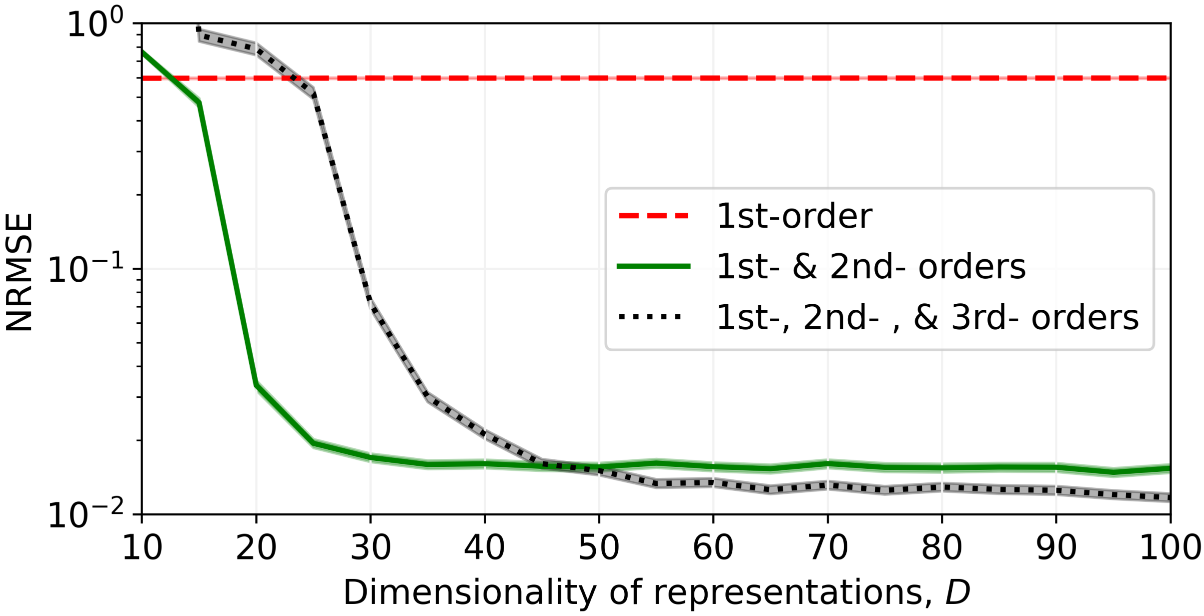

Figure S.6: **Median predictive performance for Lorenz63 system against the dimensionality of the distributed representation D when the representation is formed using features of different orders.** For each configuration, NRMSE is computed over three Lyapunov times where the reported values are obtained from 1,000 randomly initialized simulations. Shaded areas show the median standard error.

The ablation experiment in Figure S.6 demonstrates the role of choosing orders of features to be included into the distributed representation in the case of the Lorenz63 system. Note that similar results for the Mackey-Glass system were reported in the main text, Figure 4**c**. Green solid line corresponds to the line in Figure 4**a** and depicts the configuration including the first and second-order features. Importantly, when considering time-delayed states alone (red dashed line) the predictive model performs poorly demonstrating the need of including the products between the time-delayed states for obtaining the adequate performance. Inclusion of third-order features increases the minimal dimensionality of the distributed representations, but once a sufficient number of dimensions is provided, such a feature space could further improve the performance.

S-VI Performance for the Lorenz63 system with different models of vector symbolic architectures

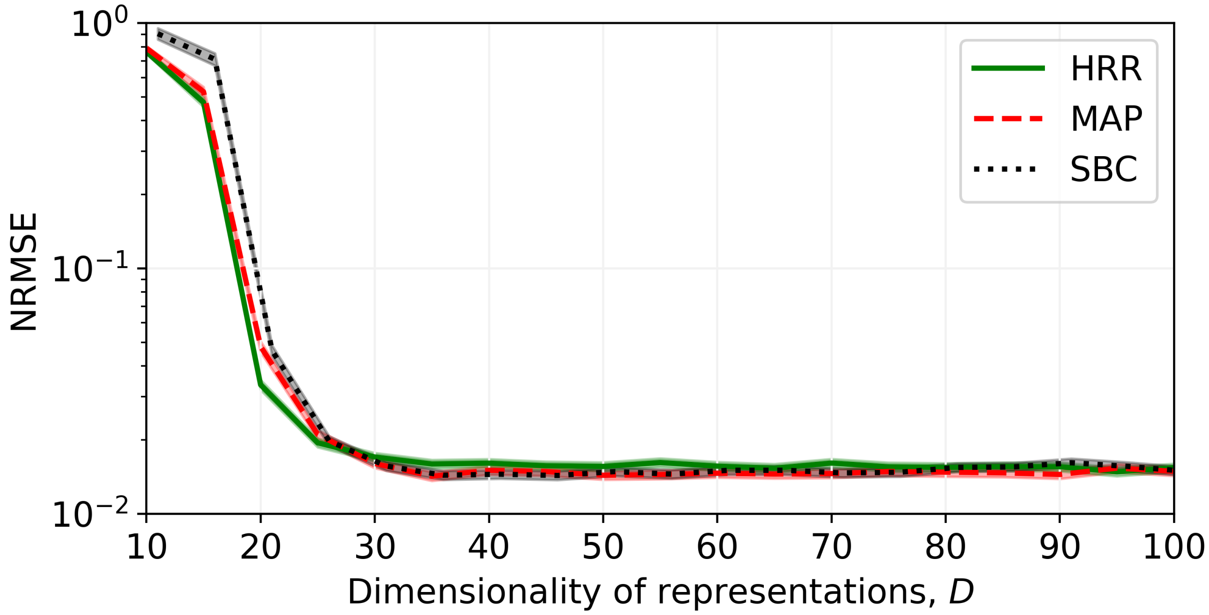

Figure S.7: **Median predictive performance for Lorenz63 system against the dimensionality of the distributed representation D for three different vector symbolic architectures.** The considered models are holographic reduced representations (HRR), multiply-add-permute model (MAP), and sparse block codes (SBC). For each configuration, NRMSE is computed over three Lyapunov times where the reported values are obtained from $1,000$ randomly initialized simulations. Shaded areas show the median standard error.

The ablation experiment in Figure S.7 is conducted to illustrate that the distributed representation scheme performs equally well irrespective of a particular vector symbolic architectures model. In particular, we show the performance for three models for which Sigma-Pi networks were discussed in Section 2.4 of the main text. Despite the differences in implementation (see, e.g., Figure 2), it is evident from the figure, all three models perform similarly.

S-VII Performance for the Lorenz63 system with permuted random projection matrix

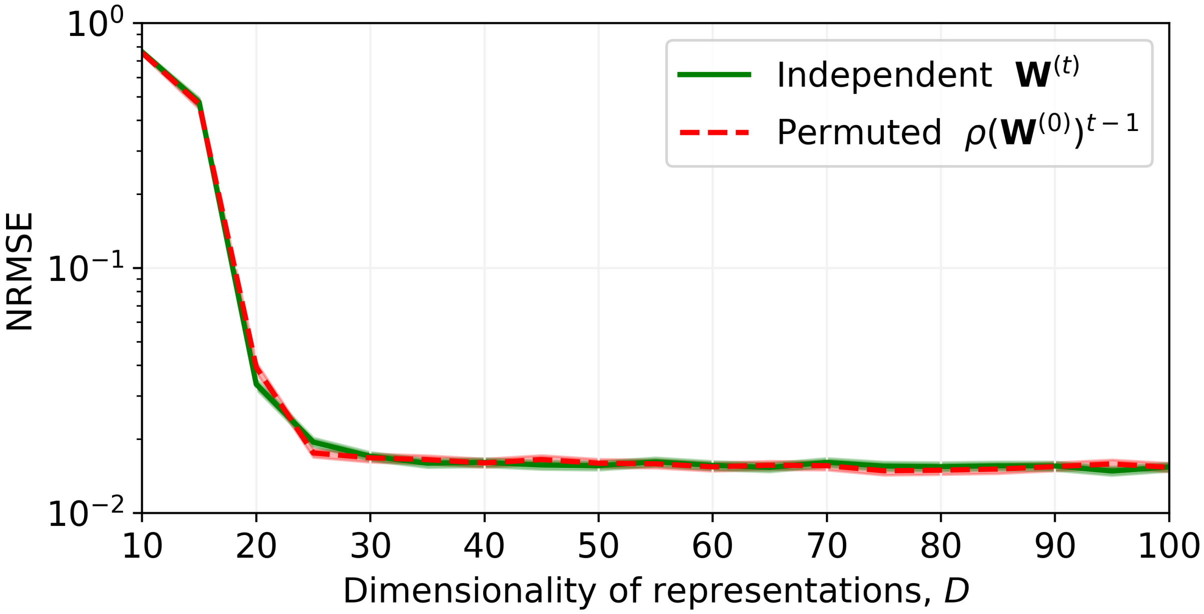

Figure S.8: **Median predictive performance for Lorenz63 system against the dimensionality of the distributed representation D for two ways of forming the distributed representation of the time-delayed states.** For each configuration, NRMSE is computed over three Lyapunov times where the reported values are obtained from $1,000$ randomly initialized simulations. Shaded areas show the median standard error.

The ablation experiment in Figure S.8 aims at supporting **Remark 1** in Section 4.4, that it is not necessary to use a separate random projection matrix $\mathbf{W}_{\texttt{in}}^{(t)}$ for each time step t since similar results can be obtained by recursively permuting one random projection matrix using a random permutation $\rho()$ where for the t-th time step $\mathbf{W}_{\texttt{in}}^{(t)} = \rho\left(\mathbf{W}_{\texttt{in}}^{(0)}\right)^{t-1}$ (cf. Eq. (17)). We implemented both variants of forming the distributed representations of first-order features. The results for the Lorenz63 system are depicted in Figure S.8 and both variants perform equally well.

S-VIII Predicting verification of the Lorenz63

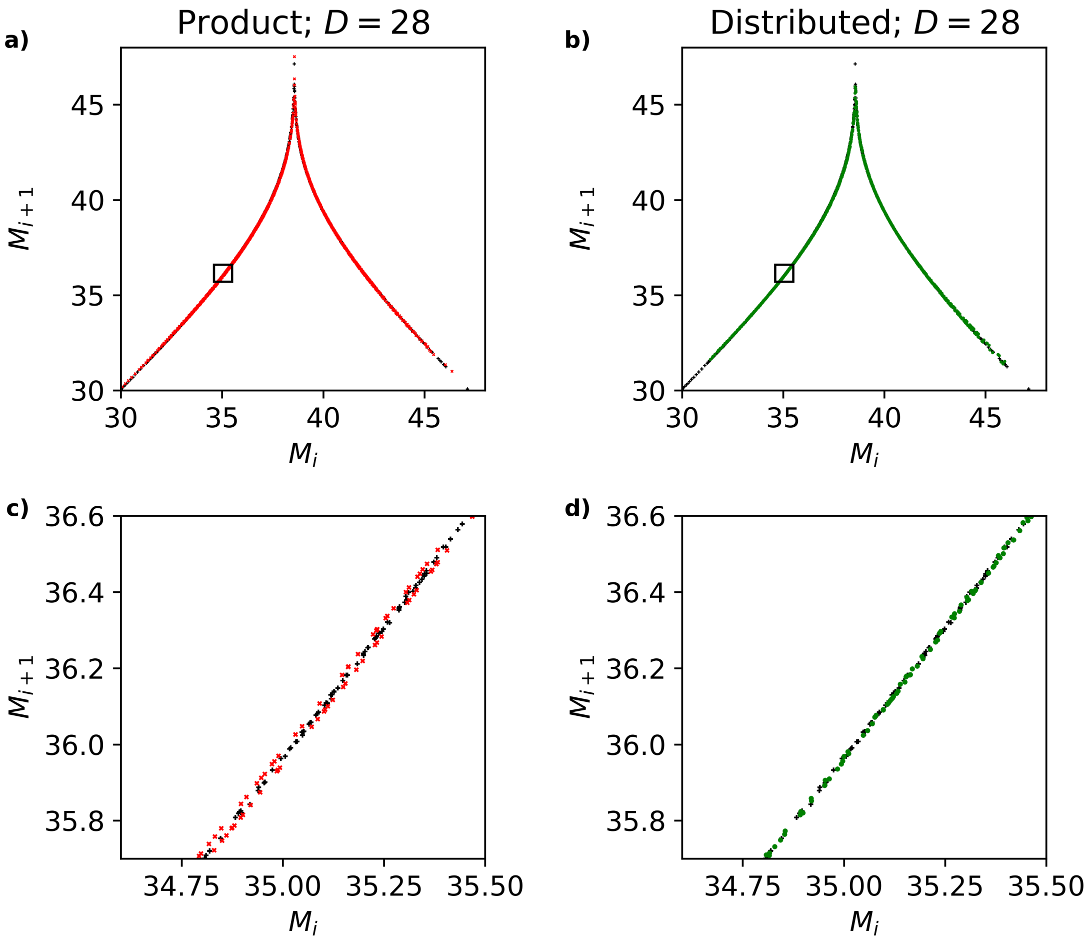

Figure S.9: [**a**) & **b**)] The z return map of the Lorenz63 system (black $+$) overlaid with z return maps of the forecasts of the product (red $\times$) and distributed (green $\circ$) representations, respectively. Both schemes reproduce the long-term dynamics of the z variable accurately enough at this scale. [**c**) & **d**)] Details of the regions marked in **a**) and **b**), respectively. The product representation: $\alpha = 2.5 \times 10^{-6}$; the distributed representation: $\alpha = 1 \times 10^{-6}$, $D = 28$. The results are obtained using $1,000$ time units in the prediction phase.

Following Supplementary Note 1 in [1], in this section we demonstrate that as for the product representation, the distributed representation predicts the attractor that is accurately representing the true one. The Lorenz63 system in Eq. (23) has three unstable steady states (USSs) determined by the following values:

$$\mathbf{X}_{\texttt{USS}} = [0, 0, 0]^\top, [\pm\sqrt{(8/3)(28-1)}, \pm\sqrt{(8/3)(28-1)}, (28-1)]^\top.$$

The predicted USSs are, thus, used to calculate the L_2 distance between them and the corresponding true USSs. In a uniformly scaled space with the unit variance, the average L_2 distance ($1,000$ simulations) from the zero USS is $1.6 \pm 0.9 \times 10^{-3}$ for the product representation and $5.3 \pm 0.5 \times 10^{-3}$ for the distributed representation while the distances between the predicted and true positive (negative) USSs are $9.6 \pm 7.5 \times 10^{-4}$ ($15.7 \pm 3.6 \times 10^{-4}$) and $10.5 \pm 3.4 \times 10^{-4}$ ($9.6 \pm 2.6 \times 10^{-4}$), respectively.

Another way to qualitatively compare the long-term behavior of the true and predicted systems for the Lorenz63 is by computing the successive local maxima M_i of state z and looking at the return map of M_i against M_{i+1}. For both schemes the maps are shown in Figure S.9 and both maps qualitatively resemble the one obtained from the ground truth.

S-IX Performance against the size of the training data

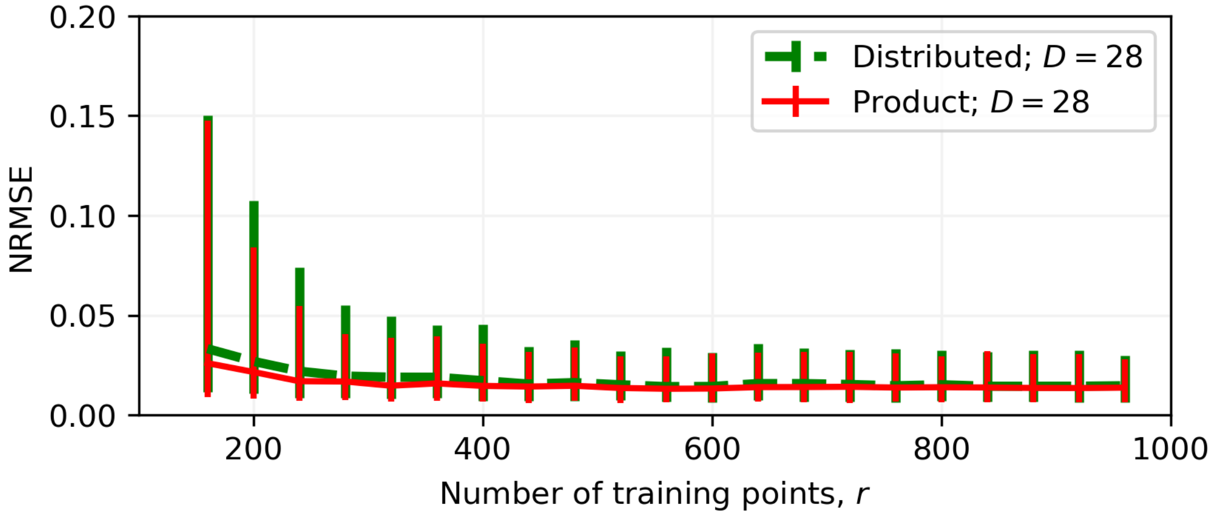

Figure S.10: **Median predictive performance of the product (red solid line) and distributed (green dashed line) representations on the Lorenz63 system as in Figure 3a-c against the size of the training data.** The error bars show 25th and 75th percentiles. For each configuration NRMSE is computed over three Lyapunov times where the reported values are obtained from $1,000$ randomly initialized simulations.

In [1], one of the advantages of the product representation over the conventional reservoir computing, which similar to our approach uses randomized representations, is much lower amount of training data necessary to produce high-quality predictions. Here, we demonstrate that the same advantage holds for the proposed approach that is similar to the reservoir computing relies on randomized representations. To do so the Lorenz63 system is used within the same setup as in Figure 3**a-c**. The following hyperparameters are used during the experiment: for the product representation $\alpha = 2.5 \times 10^{-6}$ while for the distributed representation $\alpha = 1 \times 10^{-7}$, $D = 28$. Figure S.10 shows the median NRMSE for both schemes against 21 different sizes of the training dataset that varies in the range $[160, 960]$ with step 40. For both schemes, $1,000$ simulations with random initialization of the Lorenz63 system, Eq. (23), are performed for each size. As depicted in the figure, for both schemes the values of NRMSE start to saturate at the same level – at about 300 training time points. When the training data is small both schemes show higher NRMSE with the distributed representation being slightly less stable.

S-X Performance for the Lorenz63 system driven by noise

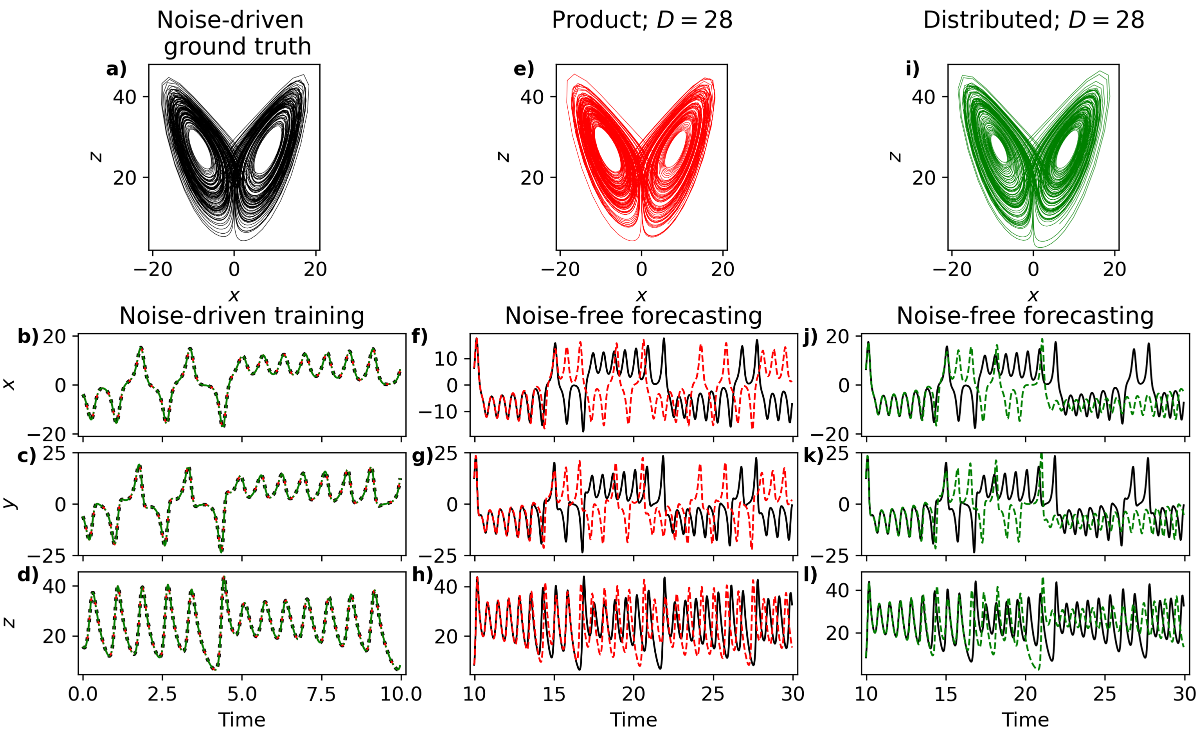

Figure S.11: **Robustness to noise.** **a**)–**d**) Noisy training data. **e**)-**l**) comparison of the noise-free ground truth behavior to predictions from the product [**e**)-**h**)] and distributed [**i**)-**l**)] representations.

This experiment aims at demonstrating that the distributed representation is as robust to noise as the product one. The experiment is the modification of the first task where noise generated from the standard normal distribution is injected into the right-hand-side of the differential equations describing the Lorenz63 system, cf. Eq. (23). To account for the noise and improve the quality of predictions, the regularization parameter for the product representation is increased to $\alpha = 1.4 \times 10^{-2}$ as opposed to $\alpha = 2.5 \times 10^{-6}$ that is used in the noiseless scenario reported in Figure 3**a-c**. For the distributed representation, the regularization parameter is also increased to 1×10^{-5}. The noisy dynamics for the training phase and the corresponding predictions of both schemes are shown in Figures S.11**a-d**. During the prediction phase, the models are initialized by the last training time point and then operate in the autoregressive mode as autonomous dynamical systems. The corresponding predictions are depicted in the central column for the product representation, Figures S.11**e-h**, and in the right column for the implicit one, Figures S.11**i-l**. When compared to the noiseless dynamics of the Lorenz63 system, it is clear that both schemes are able to learn the underlying deterministic system despite the presence of noise.

Supplementary references

[1] D. J. Gauthier, E. Bollt, A. Griffith, and W. A. S. Barbosa. Next generation reservoir computing. *Nature Communications*, 12(1):1–8, 2021.

[2] T. A. Plate. Randomly connected Sigma-Pi neurons can form associative memories. *Network: Computation in Neural Systems*, 11(4):321–332, 2000.

[3] T. Poggio. On optimal nonlinear associative recall. *Biological Cybernetics*, 19(4):201–209, 1975.
